# Efficacy of a strategy for implementing a guideline for the control of cardiovascular risk in a primary healthcare setting: the SIRVA2 study a controlled, blinded community intervention trial randomised by clusters

**DOI:** 10.1186/1471-2296-12-21

**Published:** 2011-04-19

**Authors:** Francisco Rodríguez-Salvanés, Blanca Novella, María Jesús Fernández Luque, Luis María Sánchez-Gómez, Lourdes Ruiz-Díaz, Rosa Sánchez-Alcalde, Belén Sierra-García, Soledad Mayayo, Marta Ruiz-López, Pilar Loeches, Javier López-Gónzález, Amelia González-Gamarra

**Affiliations:** 1 Unidad de Información Clínico Asistencial. Servicio de Admisión y Documentación Clínica. Hospital Universitario de la Princesa (HUP). Instituto de Investigación sanitaria del Hospital Universitario de La Princesa (IP). Red Temática de Investigación en Enfermedades Cardiovasculares (RECAVA). (C/Diego de León 62), Madrid, (28006), España; 2 Centro de Salud Potosí, Atención Primaria de Madrid, SERMAS, Consejería de Sanidad, Comunidad de Madrid. Instituto de Investigación sanitaria del Hospital Universitario de La Princesa (IP). Red Temática de Investigación en Enfermedades Cardiovasculares (RECAVA). (C/Potosí 7), Madrid, (28016), España; 3 Agencia Laín Entralgo, Consejería de Sanidad, Comunidad de Madrid. Instituto de Investigación sanitaria del Hospital Universitario de La Princesa (IP). (C/Gran Vía 27), Madrid, (28013), España; 4 Agencia de Evaluación de Tecnología Sanitarias (AETS), ISCIII. Instituto de Investigación sanitaria del Hospital Universitario de La Princesa (IP). (C/Monforte de Lemos 5), Madrid, (28029), España; 5 Centro de Salud Valleaguado, Atención Primaria Madrid, SERMAS, Consejería de Sanidad, Comunidad de Madrid. Instituto de Investigación sanitaria del Hospital Universitario de La Princesa (IP). (Avda Príncipes de España 30), Coslada, (28820), España; 6 Centro de Salud Valdebernardo, Atención Primaria Madrid, SERMAS, Consejería de Sanidad, Comunidad de Madrid. Instituto de Investigación sanitaria del Hospital Universitario de La Princesa (IP). (Bulevar Indalecio Prieto, 26), Madrid, (28032), España; 7 Centro de Salud Nuñez Morgado, Atención Primaria Madrid, SERMAS, Consejería de Sanidad, Comunidad de Madrid. Instituto de Investigación sanitaria del Hospital Universitario de La Princesa (IP).(C/Núñez Morgado s/n), Madrid, (28036), España; 8 Centro de Salud Londres, Atención Primaria Madrid, SERMAS, Consejería de Sanidad, Comunidad de Madrid. Instituto de Investigación sanitaria del Hospital Universitario de La Princesa (IP). (C/Londres 55), Madrid, (28006), España; 9 Centro de Salud San Andrés, Atención Primaria Madrid, SERMAS, Consejería de Sanidad, Comunidad de Madrid. Instituto de Investigación sanitaria del Hospital Universitario de La Princesa (IP). (C/Alberto Palacios 22), Madrid, (28021), España; 10 Centro de Salud Dr Tamames, Atención Primaria Madrid, SERMAS, Consejería de Sanidad, Comunidad de Madrid. Instituto de Investigación sanitaria del Hospital Universitario de La Princesa (IP). (Pza del Dr Tamales s/n), Coslada, 28820, España; 11 FREMAP. Instituto de Investigación sanitaria del Hospital Universitario de La Princesa (IP). (Avenida Pablo Iglesias 36-40), Madrid, (28039), España; 12 Centro de Salud Goya, Atención Primaria Madrid, SERMAS, Consejería de Sanidad, Comunidad de Madrid. Instituto de Investigación sanitaria del Hospital Universitario de La Princesa (IP). (C/O´Donnell 55), Madrid, (28009), España

**Keywords:** Primary healthcare, Randomised clinical trial, Cluster analysis, Clinical practice guidelines, Cardiovascular disease

## Abstract

This work describes the methodology used to assess a strategy for implementing clinical practice guidelines (CPG) for cardiovascular risk control in a health area of Madrid.

**Background:**

The results on clinical practice of introducing CPGs have been little studied in Spain. The strategy used to implement a CPG is known to influence its final use. Strategies based on the involvement of opinion leaders and that are easily executed appear to be among the most successful.

**Aim:**

The main aim of the present work was to compare the effectiveness of two strategies for implementing a CPG designed to reduce cardiovascular risk in the primary healthcare setting, measured in terms of improvements in the recording of calculated cardiovascular risk or specific risk factors in patients' medical records, the control of cardiovascular risk factors, and the incidence of cardiovascular events.

**Methods:**

This study involved a controlled, blinded community intervention in which the 21 health centres of the Number 2 Health Area of Madrid were randomly assigned by clusters to be involved in either a proposed CPG implementation strategy to reduce cardiovascular risk, or the normal dissemination strategy. The study subjects were patients ≥ 45 years of age whose health cards showed them to belong to the studied health area. The main variable examined was the proportion of patients whose medical histories included the calculation of their cardiovascular risk or that explicitly mentioned the presence of variables necessary for its calculation. The sample size was calculated for a comparison of proportions with alpha = 0.05 and beta = 0.20, and assuming that the intervention would lead to a 15% increase in the measured variables. Corrections were made for the design effect, assigning a sample size to each cluster proportional to the size of the population served by the corresponding health centre, and assuming losses of 20%. This demanded a final sample size of 620 patients. Data were analysed using summary measures for each cluster, both in making estimates and for hypothesis testing. Analysis of the variables was made on an intention-to-treat basis.

**Trial Registration:**

ClinicalTrials.gov: NCT01270022

## Background

Clinical practice guidelines (CPGs) can be defined as a series of systematically developed recommendations designed to help health professionals and patients take decisions regarding the most appropriate forms of treatment in specific clinical situations [1]. They are used with the aim of preventing variability in clinical practice via the presentation of the best scientific evidence [2]. This, however, should not be their only aim [3]; they should also be used to solve organisational problems, adjusting their recommendations to the preferences and values of patients, the availability of resources, and the concerns of managers [4]. A great many CPGs have been produced in recent years. In 1992 the National Library of Medicine of the USA published an exhaustive monograph recounting the appearance of 533 between the years of 1985 and 1992 [5], yet this can only be an approximation to their true number. In Spain, the *Guía Salud *Project collected and reviewed 425 CPGs in February 2010 [6].

The evaluation of the use of CPGs in terms of health results is not very extended in Spain. With the exception of the *Programa de Actividades Preventivas y de Promoción de la Salud *(PAPPS; Program of Preventive Activities and Health Promotion) [7], no studies on the effectiveness of CPGs at the primary healthcare level appear to exist. A systematic review published in the Lancet in 1993 [8] reported that, of 59 reviewed CPGs, none of which were used in the primary healthcare setting, 55 had improved the health of patients following their implantation. This improvement appeared to be related to the implementation strategy used. Studies that have assessed the impact of CPGs in the primary healthcare setting, however, show them not to have been of benefit to patients. A review focused on primary healthcare that investigated 91 studies found only five interventions to have been of any benefit to patients [9]. In fact, only 13 actually met the inclusion criteria of the study. The authors of this review warn of the poor quality of the CPGs in question and their scant implementation.

According to a review of studies on the implementation of CPGs, only 40% of health professionals actually read them, and while 78% profess to use them the real figure could be as low as 5% [10].

It can be inferred that the use a CPG is related to a number of concatenated factors: it would appear important that health professionals relate to the CPG in question [11], that local adaptations have been made involving people close to the potential end users, and that guidelines are adjusted to the peculiarities of the setting in which they are to be used. These factors facilitate the implementation of CPGs, improve their use, and increase their final impact [12]. However, one of the most important factors affecting the use of CPGs is the implementation strategy employed [13]. No evidence is available, however, on what night be the best implementation strategy. The classic review by Fremantle [14] reports that the implementation of CPGs based on educational methods and using computer-based reminders can improve use by some 15%. Certainly, however, no studies are available on what might be the best way to implement a CPG at the primary healthcare level in Spain.

All bodies that propose recommendations for the production of CPGs indicate that implementation strategies should be included in the document's planning. Failure to do this is unlikely to lead to any changes in medical practice. The diffusion methods that have given the best results have been those designed to bring about a positive predisposition in the end user before the CPG is presented, e.g., via the release of part of the information so that end users might consider the need for change, and offering them alternatives in the use of the CPG, assuring them that it contains no strict rules to follow but recommendations that the professional should use adequately with each individual patient [15].

None of the above studies, however, affords conclusive results. In this light, the present work describes the aims and design of a project undertaken to determine the effectiveness of a CPG implementation strategy for the control of cardiovascular risk. A CPG for the detection and treatment of this problem was chosen given the important public health burden of heart disease, and because, despite the potential benefits of using CPGs in this area, their true use by primary care practitioners has room for improvement [16].

## Aims

The main aim of this work was to compare the effectiveness of 1) a proposed strategy based on an educational method involving the participation of opinion leaders with 2) the usual method of dissemination, for implementing a locally adapted CPG for the control of cardiovascular risk among the primary healthcare teams of a health area in Madrid.

## Methods

This study (ClinicalTrials.gov Identifier number: NCT01270022) was a controlled, blinded community intervention study, randomised by clusters (primary healthcare centres). This design was chosen since the intervention necessarily involved primary healthcare teams. Twenty one health centres of the Madrid A2 Primary Healthcare Area were randomly assigned to the normal dissemination or proposed implementation strategy arms of the study (10 to the former and 11 to the latter). These 21 health centres (clusters) provide care to 373,495 people. The two strategies were aimed at the healthcare teams (doctors and nurses) of each participating centre. The effect of the two strategies on 1) the recording of cardiovascular risk data in the clinical histories of the patients, 2) the control of cardiovascular risk factors, and 3) the incidence of cardiovascular events, was determined.

### Intervention

The CPG used in this study was a local adaptation of the different cardiovascular risk guidelines established by healthcare professionals working in both the primary and hospital settings. The dissemination of the CPG to the normal dissemination group was via the method traditionally used in the health area, i.e., sending it by mail to all healthcare professionals and the provision of an information workshop lasting 2 h. The strategy used with the implementation group involved an educational model based on the involvement of opinion leaders *plus *the method used with the normal dissemination group. The implementation strategy, which was aimed at the doctors and nurses employed at the health centres, involved 4 × 1 h education sessions. These were masked within the normal staff meeting held at each centre to avoid the undertaking of the present study becoming known. The sessions were led by trained personnel recognised as leaders of opinion by the healthcare professionals at the centres - a normal practice in these session. The first session was delivered by a doctor and a nurse, members of the team that coordinated the authoring of the CPG. During this session the methodology used to produce the CPG was explained, underlining the importance of stratifying the cardiovascular risk of patients attending primary healthcare clinics. In the second session, a nurse from the health area (one of the authors of the CPG) discussed with the health centre staff the diagnosis, classification and treatment of low risk patients, screening techniques for different cardiovascular risks, and methods for evaluating these risks. In the third session, a physician author of the CPG with prestige amongst his/her peers discussed the pharmacological recommendations associated with cardiovascular risk factors, and the diagnosis, treatment and classification of high risk patients. In the final session, a physician author (an internal medicine specialist) from the *Hospital Universitario de la Princesa*, discussed referral criteria and the coordination proposals included in the CPG.

### Study stages

The study was divided into three stages. 1) *Preliminary work*. In July 2004, a cross-sectional study was undertaken to determine the status of the cardiovascular information in the selected patients' records. This involved the collection of the data required to determine the different risk factors recorded, the degree of control of cardiovascular risk factors, and the collection of other information required for the present study. 2) *The intervention*. This intervention took place between February and April 2005. One year later (April 2006) a second cross-sectional study was undertaken to determine the effect of the two strategies on the calculation of cardiovascular risk, and the control of these risk factors. 3) *Follow-up*. This phase involved a third cross-sectional study to determine the effect of the intervention on health results (cardiovascular events and the control of cardiovascular risk factors) at five years.

### Data collection

**Figure 1:**
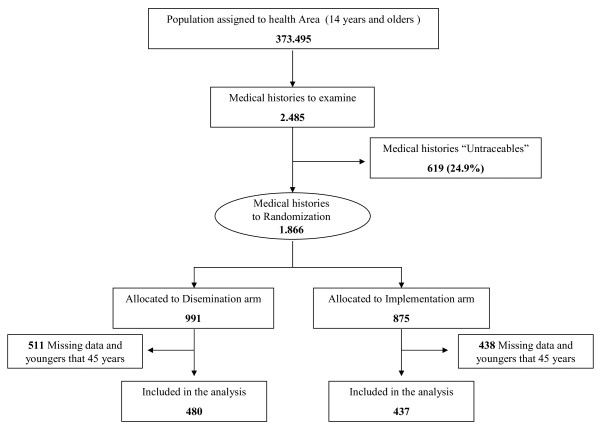
**Patients Flow chart: number of patients finally studied and their distribution**.

The medical records of all patients aged 14 years and older who according to their medical cards, belonged to the studied health area, were collected. The initial subjects to be included were selected by random sampling weighted by the size of the population assigned to each health centre (Figure 1). This step was undertaken to ensure that the final sample would be independent of the aging of the population of each centre. Among these selected subjects, those aged 45 years and over were chosen to be the final study subjects. This age was chosen since it is that given priority in community intervention studies on cardiovascular risk, both in men and women [17]. Patients with no medical history available were classified as non-locatable. Those patients whose medical histories did not mention their age or sex were excluded, as were those who had not visited their health centre in the previous year.

To ensure blindness in the assessment of the effectiveness of the intervention (evaluation of the above variables in clinical records), the evaluators were not told to which arm of the study the patients belonged. The team members whose job it was to analyse the results did not know this information either.

### Variables measured

The impact of the intervention was measured in terms of registry variables (cardiovascular risk data in the clinical histories of the patients), intermediate variables (the control of cardiovascular risk factors at one and five years) and health results (the incidence of cardiovascular events at five years). At one year, the effect of the two strategies was determined by calculating the difference in the proportions of overall cardiovascular risk calculations before and after the intervention, or the explicit recording of all the variables needed for such calculations to be made (blood pressure, fasting glycaemia, fasting cholesterol, use of tobacco, age and sex). Blood pressure was deemed well controlled when the systolic pressure was ≤ 140 mmHg and the systolic blood pressure ≤ 90 mmHg; this was reduced to 130/80 mmHg if the patient was diabetic. Good control of glycaemia was deemed present when fasting blood sugar was < 126 mg/dl. The control of dyslipidaemia was considered good when total cholesterol was < 200 mg/dl, or LDL-cholesterol was < 100 mg/dl in diabetics.

At five years, the differences in health variables between the arms of the study, i.e., percentage of cardiovascular events (acute myocardial infarction, angina, stroke and the need for revascularization), the cardiovascular mortality and overall mortality, were determined. Since each of the health centres had different characteristics, data on a series of potential confounding factors (characteristics of the health centre and their staff) were recorded.

### Sample size

The unit of randomisation taken into account was not each patient but each health centre. The number of centres included in the study was 21 - all those in the studied health area. The sample of patients finally selected at any centre was proportional to the size of the population ascribed to that centre. The required sample size was determined by assuming that the implantation of the CPG could be regarded as successful if a 15% increase in the recording of the study variables were seen in the proposed implementation strategy arm, i.e., increasing from 40% to 55%. To show this effect, the sample size needed to reject the null hypothesis could have been calculated in the normal way [18] for the comparison of two proportions. For an alpha of 0.05 and beta of 0.20, the necessary sample size would have been n = 173*2 = 346 patients. However, designs involving randomisation by clusters with a fixed number of clusters requires a larger sample in order to take into account the design effect (DE). According to Campbell [19], DE = 1 + (m - 1) · r, where m is the mean size of the cluster and r the intracluster correlation coefficient (ICC). Since 346 patients would be needed if sampling were not via clusters, and since the study involved 21 health centres, the mean sample size per cluster (m) (if clusters are used) becomes 16.5 (346 patients across 21 centres).

Given that reported by Adams et al. [20], an ICC of 0.032 was assumed for the majority of variables examined. The DE therefore became: DE = 1 + (m-1) · r = 1.5. The corrected sample size was therefore: n´= n · ED = 346 · 1.5 = 517. Assuming a loss rate of 20%, the final sample size would be 620 patients randomly assigned to the two arms of the study (10 clusters in the normal dissemination group, and 11 in the proposed implementation strategy group). This sample size was adjusted for the fact that, in the studied health area, some 47% of the population is over 45 years of age. The total number of medical histories to examine was therefore at least 1170 for both arms together. Figure 1 shows the number of patients finally studied and their distribution.

### Statistical analysis

The use of a design involving randomisation by clusters conditions the statistical methods that can be used. Although some authors [21] propose performing individual level analysis when the number of clusters is >20, there is no consensus [22], and since the present number was 21 a more conservative approach (cluster level analysis) was followed. The overall impact of the intervention was determined by comparing the means of the variables measured in each cluster using the t-test. Although the assumptions of homoscedasticity and normal distribution may not be satisfied, this is not so important since the t-test is robust to such departures [23]. Since the cluster sizes were not similar, the t-test was weighted for each cluster j by wj = nj1·nj2/nj1+nj2 (where 1 and 2 are moments before and after the intervention respectively) [24]. The calculation of the confidence intervals also required weighting in the same way. Analysis was on an intention-to-treat basis. All p values reported were two-tailed. STATA v.8.0 software was used for all calculations.

## Discussion

Cardiovascular risk was chosen as an indicator of change for three reasons: its measurement is essential in the reduction of cardiovascular events, its measurement depends completely on good practice on the part of medical professionals, and it is not influenced by patient attitude or any medical professional/patient or patient/healthy system relationship. The present strategy is relatively simple and, if shown to provide reliable results, could be used in other settings in which the use of more complex designs (see for example 25) may not be viable.

A number of reasons dictated that randomisation by clusters be used rather than simple randomisation, despite the lower statistical power of this method [26]. Firstly, since all the healthcare staff of each centre were involved, it avoids the contamination that might occur if simple randomisation were followed. Secondly, although the intervention was aimed at the staff, the assessment took the form of an audit of the patients' clinical records. Finally, since randomisation was by clusters, analysis by clusters was therefore necessary. In recent years, the method employed in this work has become standard in the assessment of the effects of health interventions (implementation research) [27].

## List of abbreviations used

CPG: Clinical Practice Guidelines; DE: Design Effect; ICC: Intracluster Correlation Coefficient

## Ethical concerns

The described trial was approved by the *Comisión de Investigación del Área *2. Consent to be included was obtained from all the health centres involved. Medical histories were consulted only by the research team. At all times confidentiality was preserved; no patients or medical professionals were identified.

## Competing interests

The authors declare that they have no competing interests.

## Authors' contributions

BNA, RSA, and LRD had the original idea for the study.

BNA, FRS and LMSG designed the protocol and wrote the drafts of the paper.

MJFL, LMSG, LRD, RSA, BSG, AGG, PLB, SMV, MRL and JLG participated writing the drafts.

BNA is the author responsible for the study and coordinated the research group.

All the authors have read and approved the final manuscript.

## Pre-publication history

The pre-publication history for this paper can be accessed here:

http://www.biomedcentral.com/1471-2296/12/21/prepub
